# The autonomic innervation of hairy skin in humans: an *in vivo* confocal study

**DOI:** 10.1038/s41598-019-53684-3

**Published:** 2019-11-18

**Authors:** Vincenzo Donadio, Alex Incensi, Veria Vacchiano, Rossella Infante, Martina Magnani, Rocco Liguori

**Affiliations:** 1 IRCCS Istituto delle Scienze Neurologiche di Bologna, UOC Clinica Neurologica, Bologna, Italy; 2 Dipartimento di Scienze Biomediche e Neuromotorie, Università di Bologna, Bologna, Italy

**Keywords:** Autonomic nervous system, Diagnostic markers

## Abstract

The autonomic innervation of the skin includes different subsets of adrenergic and cholinergic fibers both in humans and animals. The corresponding chemical code is complex and often difficult to ascertain. Accordingly, a detailed histochemical description of skin autonomic fiber subtypes is lacking in humans. To characterize skin autonomic nerve subtypes may help to better understand the selective damage of specific skin autonomic fibers affecting human diseases such as the adrenergic fibers directed to skin vessels in Parkinson’s disease or the cholinergic sudomotor fibers in Ross Syndrome. The present study aimed at characterizing subtypes of autonomic fibers in relation to their target organs by means of an immunofluorescent technique and confocal microscopy. We studied 8 healthy subjects (5 males and 3 females) aged 45 ± 2 (mean ± SE) years without predisposing causes for peripheral neuropathy or autonomic disorders. They underwent skin biopsy from proximal (thigh) and distal (leg) hairy skin. A combination of adrenergic (i.e. tyrosine-hydroxylase- TH and dopamine beta-hydroxylase- DbH) and cholinergic (vesicular acetylcholine transporter- VACHT) autonomic markers and neuropeptidergic (i.e. neuropeptide Y- NPY, calcitonin gene-related peptide- CGRP, substance P- SP, and vasoactive intestinal peptide- VIP) markers were used to characterize skin autonomic fibers. The analysed skin autonomic structures included: 58 sweat glands, 91 skin arterioles and 47 arrector pili muscles. Our results showed that all skin structures presented a sympathetic adrenergic but also cholinergic innervation although in different proportions. Sympathetic adrenergic fibers were particularly abundant around arterioles and arrector pili muscles whereas sympathetic cholinergic fibers were mainly found around sweat glands. Neuropeptides were differently expressed in sympathetic fibers: NPY were found in sympathetic adrenergic fibers around skin arterioles and very seldom sweat glands but not in adrenergic fibers of arrector pili muscles. By contrast CGRP, SP and VIP were expressed in sympathetic cholinergic fibers. Cholinergic fibers expressing CGRP, SP or VIP without TH or DbH staining were found in arterioles and arrector pili muscles and they likely represent parasympathetic fibers. In addition, all skin structures contained a small subset of neuropeptidergic fibers devoid of adrenergic and cholinergic markers with a likely sensory function. No major differences were found between males and females and proximal and distal sites. In summary hairy skin contains sympathetic adrenergic and cholinergic fibers differently distributed around skin structures with a specific distribution of neuropeptides. The autonomic skin innervation also contains a small amount of fibers, likely to be parasympathetic and sensory.

## Introduction

The autonomic innervation of hairy skin includes different subsets of adrenergic and cholinergic fibers both in humans and animals but the chemical code of autonomic nerve terminals is complex and not well known^1–4^. This is unfortunate since the neuropeptide content of a fiber subpopulation is likely to define important aspects of its functional characteristics. Other such aspects are related to the firing characteristics of the efferent nerve fibres (which can be studied by microneurography^5,6^) but the link between the neurochemical codes of efferent C fiber populations and their firing patterns is unknown making the interpretation of frequently used autonomic tests difficult^7,8^. A better knowledge of these factors and relationships may also lead to a better understanding of the selective damage of autonomic fibers in human diseases, such as Parkinson´s disease^9–11^ or Ross syndrome^12,13^.

Against this background the present study aimed to neurochemically characterize subtypes of skin autonomic fibers in relationship to their target organs, using a combination of adrenergic, cholinergic and neuropeptidergic markers by means of an immunofluorescent technique and confocal microscopy.

## Subjects and Methods

We studied 8 healthy subjects (5 males and 3 females) aged 45 ± 2 (mean ± SE) years, reporting no predisposing causes for peripheral neuropathy or autonomic disorders, such as diabetes, microbiological, autoimmune, paraneoplastic and thyroid diseases.

The procedures we used complied with the Helsinki Declaration regarding international clinical research involving human beings. The local Human Ethics Committee (Comitato Etico di Area Vasta Emilia Centro della Regione Emilia-Romagna, CE-AVEC, Bologna, N° 122/2018) approved the study and all subjects gave their written informed consent to the study.

### Skin biopsy

Following a previously described protocol^14,15^ 3 mm punch biopsies were taken from the thigh (15 cm above the patella) and the distal leg (10 cm above the lateral malleolus). Skin samples were immediately fixed in cold Zamboni’s fixative and kept at 4 °C overnight. Fifty μm sections were obtained using a cryostat (HM550, Thermo Scientific, Walthan, MA, USA). Free-floating sections were incubated overnight with a panel of primary antibodies listed in Table 1. In short, primary antibodies included the pan-neuronal marker protein gene product 9.5, a specific marker for collagen IV to identify the structure of skin annexes, autonomic markers both for adrenergic fibers such as tyrosine-hydroxylase (TH) and dopamine beta-hydroxylase (DbH) and cholinergic fibers like vesicular acetylcholine transporter (VACHT). Primary antibodies to identify neuropeptides usually co-expressed in autonomic nerve fibers endings included the vasoactive intestinal peptide (VIP), calcitonin gene-related peptide (CGRP), neuropeptide Y (NPY), and substance P (SP). Sections were then washed and secondary antibodies labelled with mouse or rabbit Alexa Fluor(R) 488 (1:400; Jackson ImmunoResearch, West Grove, PA, USA), goat cyanine dye fluorophores 3.18 (1:400, Jackson ImmunoResearch, West Grove, PA, USA) and mouse or rabbit 647 (cyanine dye fluorophores 5, 1:400, Abcam, Cambridge, UK) were added for overnight incubation. Digital images were acquired using a laser-scanning confocal microscopy and subsequently projected to obtain a digital image by a computerized system (Nikon confocal microscopy, Eclipse Ti A1). The sections selected for the analysis include frames of 0.5 μm on a Z-stack plan at the appropriate wavelengths for secondary antibodies with a x400 or x600 plan apochromat objective. The co-localization between two or three different fluorescent signals was first judged absent or present on a single 0.5 μm frame by the agreement of two authors with major experience of immunoflorescence analysis (DV and IA).

**Table 1:** List of primary antibodies used.

Antibody	Host	Dilution	Producer	Cat. number
PGP9.5	rabbit	1:500	Abcam	ab108986
PGP9.5	mouse	1:750	Abcam	ab72911
Collagen IV	mouse	1:1000	Millipore	MAB1910
VIP	rabbit	1:1000	Immunostar	20077
VIP	mouse	1:200	Novus Biol.	NBP1-05163
VaChT	goat	1:500	Santa Cruz	Sc-7717
DßH	rabbit	1:150	Millipore	AB1536
TH	rabbit	1:1000	Novus Biol.	NB300-109
TH	goat	1:500	Santa Cruz	Sc-7847
NPY	rabbit	1:1000	Abcam	ab30914
SP	rabbit	1:1000	Immunostar	20064
SP	mouse	1:500	Santa Cruz	Sc-58591
CGRP	rabbit	1:500	Immunostar	24112
CGRP	mouse	1:750	Santa Cruz	Sc-57053

PGP 9.5 = protein gene product 9.5; VIP = vasoactive intestinal peptide; VaChT = vesicular acetylcholine transporter; DßH = Dopamine beta-hydroxylase; TH = tyrosine-hydroxylase; NPY = neuropeptide Y; SP = substance P; CGRP = calcitonin gene-related peptide.

The characterization of autonomic fibers was made in all subjects considering 91 different skin arterioles, 47 arrector pili muscles and 58 sweat glands.

## Results

### Adrenergic innervation

Adrenergic fibers were identified by DbH staining mainly found around skin arterioles and arrector pili muscles and less around sweat glands.

**Figure 1:**
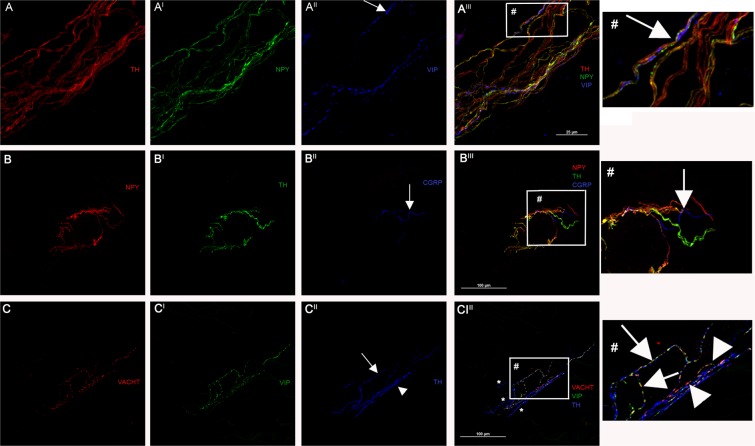
Autonomic innervation of skin arterioles. Leg autonomic innervation disclosed by confocal microscope (x600 in A and x400 in **B** and **C**). (**A**) Adrenergic fibers disclosed by a staining against TH (red) and NPY (green). They run longitudinally to the main axis of the vessel often clinging to it forming a mesh-like network. These fibers likely presented a vasoconstrictor activity considering the close and enveloping relationship with the vessel wall. A few fibers are stained by TH and VIP (blue; arrow) but not by NPY. They are likely to be sympathetic cholinergic fibers (see also **C**). These latter run close to adrenergic NPY fibers suggesting a coordinated functional activity. The strict correlation between sympathetic cholinergic and adrenergic fibers is showed in a high magnification image (#); (**B**). Around skin arterioles a few non-adrenergic fibers expressing peptidergic markers are found with a likely somatic function. In such arterioles a fiber selectively stained CGRP (blue) without TH (green) and NPY (red) staining is showed by the arrow. (**C**) Sympathetic cholinergic fibers (arrows) were stained by TH (blue), VIP (green) and VACHT (red) more clearly reported in the high resolution image (#). By contrast fibers stained only by TH were adrenergic (asterisks). A few VIP and VACT positive fibers were not stained by TH staining, which makes it likely that they represent parasympathetic fibers (head of arrow) better reported in the high resolution image (#).

*Skin arterioles* were easily recognized because of a thick collagen wall. The majority of nerve fibers innervating arterioles expressed TH, DbH and NPY immunoreactivity. These fibers were running longitudinally to the main axis of the vessel, often clinging to it forming a mesh-like network (Fig. 1A, AI). A few TH fibers without DbH expressed VIP and VACHT often together with CGRP and SP and showed a similar mesh-like pattern (Fig. 1C).

**Figure 2:**
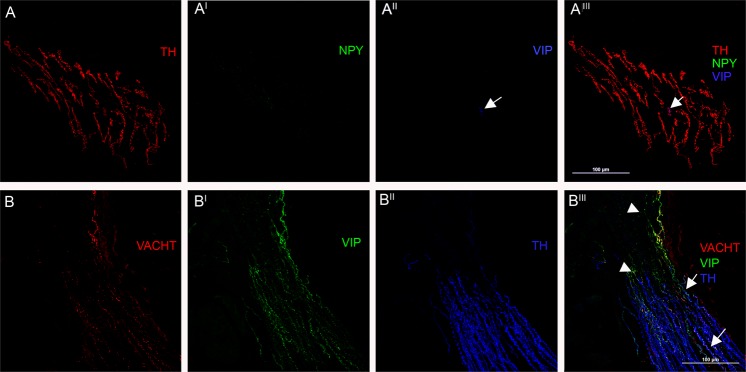
Autonomic nerves in Arrector pili muscles. Confocal microscope analysis (x400) of arrector pili muscle. (**A**) Nearly all of these fibers are adrenergic showing a staining against TH (red). Interestingly these fibers did not express NPY (green), which is different from the adrenergic fibers innervating arterioles that co-expressed NPY. This arrector pili muscle also shows a VIP positive fiber (arrows) in blue. This fiber co-expressed TH and represents a sympathetic cholinergic fiber. (**B**) Different sympathetic cholinergic fibers co-expressing TH (blue), VIP (green) and VACHT (red) staining can be seen in this arrector pili muscle (arrows). A few VIP fibers did not express TH and VACHT (head of arrows) and they are likely to be somatic fibers.

*Arrector pili muscles*. The majority of nerve fibers were adrenergic and expressed TH and DbH but in contrast to the vessels they did not present NPY (Fig. 2A; Table 2). Few TH fibers without DbH presented VIP and VACHT and usually CGRP and SP staining and they ran close to the adrenergic fibers (Fig. 2B).

**Table 2:** Percentage of nerve fibers immunoreactive for a specific marker in autonomic skin structures.

IR positivity	Skin Arterioles (91)	Arrector pili muscles (47)	Sweat glands (58)
DBH	100%	100%	10%
TH	100%	100%	95%
NPY	100%	0	0
VACHT	89%	45%	100%
VIP	93%	60%	100%
SUBP	97%	62%	85%
CGRP	98%	65%	98%

IR = immunoreactive; the number in brackets represents the number of analysed structures.

*Sweat gland*. TH and DbH positive fibers co-expressing NPY were occasionally found around a few sweat gland tubules (Table 2).

### Cholinergic innervation

Cholinergic fibers expressing VACHT were mainly found around sweat glands and less in skin arterioles and arrector pili muscles.

*Skin arterioles*. The majority of cholinergic fibers co-expressed TH and represent sympathetic cholinergic fibers. They were usually running close to adrenergic fibers (i.e. stained by DbH and NPY), suggesting a strict functional coupling (Fig. 1C). A subgroup of these fibers (usually running in the wall of vessels) did not express TH. They probably represent parasympathetic fibers with a specialized vasodilator function (Fig. 1C).

*Arrector pili muscles*. A few sympathetic cholinergic fibers expressing TH and CGRP and/or SP were running close to adrenergic fibers. Their VACHT staining was usually less intense and irregular than that of sweat gland fibers (Fig. 2B).

**Figure 3:**
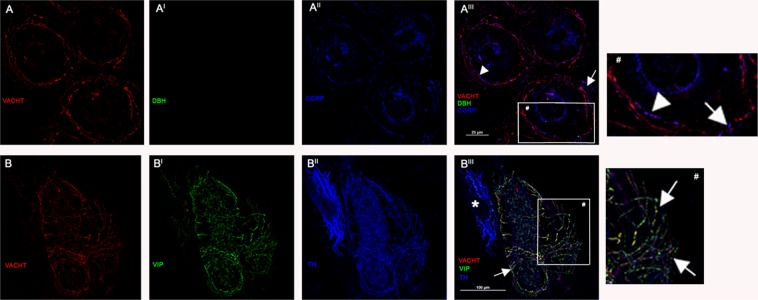
Sympathetic innervation of a sweat gland. Sympathetic innervation of a sweat gland disclosed by a confocal microscope analysis (x600 in A and x400 in B). (**A**) Cholinergic VACHT positive fibers (red) encircling sweat gland tubules. These fibers did not show a staining against DbH (although occasionally adrenergic fibers could be found around sweat tubules). The majority of these fibers co-expressed CGRP (blue) indicated by head of arrows as more clearly reported in the high resolution image (#). A few CGRP fibers that did not express VACHT (arrows) probably represent somatic fibers enlarged in the high magnification image (#). (**B**) However, the majority of sudomotor fibers are sympathetic cholinergic showing TH (blue) and VACHT (red), VIP (green) positive staining (arrows) as showed clearly showed in the high resolution image (#). These fibers encircle sweat tubules with a sudomotor activity. TH staining is less intense than sympathetic adrenergic fibers of a skin arteriole (asterisk) close to the sweat gland. In addition, the VIP and VACHT staining of the arteriole shows an interrupted staining with varicosities located at regular intervals along the nerve fiber. This is slightly different from the VIP and VACHT staining found around the sweat gland characterized by a more regular signal. These divergences may probably indicate a different concentration of the molecular machine needed for the cholinergic transmission with higher concentration in the sudomotor fibers.

*Sweat glands*. Abundant VACHT and VIP positive fibers were found around sweat tubules. Often these fibers co-expressed TH although the staining was less intense than that of adrenergic fibers of skin arterioles (Fig. 3B). Cholinergic fibers often co-expressed CGRP and SP but most commonly they were associated with CGRP (Fig. 3A; Table 2).

### Sensory function

Nerve fibers expressing VIP, CGRP and/or SP without adrenergic (i.e. TH or DbH) and cholinergic (VACTH) staining were found in all structures studied with different rate of positivity (Table 2). They represented a small contingent of fibers with a likely afferent (i.e. sensory) function (Fig. 1B).

## Discussion

The main findings of this study in healthy subjects were that hairy skin shows a complex autonomic innervation pattern with the following characteristics: (1) sympathetic adrenergic and cholinergic fibers were found in all dermal structures whereas only few parasympathetic fibers are recognized in skin arterioles and arrector pili muscles; (2) sympathetic and parasympathetic fibers co-expressed specific and different neuropeptides; (3) sensory nerves have been found in all autonomic structures that are likely to be involved in reflex regulation mechanisms.

### Skin arterioles

A previous study^16^ showed cholinergic and adrenergic fibers around arterioles involved in thermoregulation. Both types of fibers also contain several neuropeptides such as CGRP, VIP and NPY, which are potent vasoactive substances^17^. It has been demonstrated that adrenergic fibers containing NPY are vasoconstrictors^18–20^. This conclusion was also supported by the intradermal perfusion of exogenous NPY that produced cutaneous vasoconstriction^21^. Our present study showed that adrenergic fibers immunoreactive for TH, DbH and NPY are located in a network-like mesh around arterioles. Presumably, these fibers are the ones inducing the vasoconstrictor activity previously described^18–22^. However, as described in the rat and the monkey^23^ skin arterioles are also innervated by cholinergic fibers expressing VACHT without adrenergic markers (i.e. TH or DbH). They probably represent parasympathetic fibers co-expressing several neuropeptides such as VIP, CGRP or SP and they run close to adrenergic fibers with a likely antagonistic, probably vasodilator function^24,25^. In agreement with this hypothesis VIP, CGRP and SP peptides have been reported to have a vasoactive function either directly^17,26^ or by favouring the release of the endothelium-derived nitric oxide^27^. Furthermore, the presence of parasympathetic fibers in the skin arterioles was demonstrated by the persistence of VACHT positive fibers after sympathectomy that produced the disappearance of DbH fibers in rat^23^. In addition, a vasodilatation was demonstrated after direct stimulation of sympathetic fibers in the human foot^28^. A subgroup of cholinergic fibers also express TH as an indication of a persisting noradrenergic trait, likely representing sympathetic cholinergic fibers, previously described in association with arteriovenous anastomoses (Hoyer-Grosser organs) in rats^16^. The specific function of these sympathetic neurons innervating skin arterioles is unknown, we found a close anatomical proximity with adrenergic fibers suggesting a likely specialized function in regulating vasoconstriction activity.

*Sweat glands* are mainly innervated by sympathetic cholinergic fibers as suggested by the persistence of an adrenergic trait with the co-expression of TH^25^. We described that these fibers encircled sweat gland tubules likely inducing a sudomotor activity. The TH staining expressed in cholinergic fibers is less intense than that found in adrenergic fibers around skin arterioles (see Fig. 3B). This finding could be explained by the fact that these fibers presented a persistence of a previous adrenergic tract but they do not contain the appropriate metabolic machine for the noradrenergic activity. Accordingly, the amount of TH is lower than that found in a functionally working adrenergic fiber. However, this finding also suggests that TH could not be considered as a specific marker to differentiate sympathetic adrenergic from cholinergic fibers in skin nerves. Our data supported previous findings showing that neuropeptides (i.e. VIP but also CGRP) were largely co-localized in the sympathetic cholinergic sudomotor fibers^26,29^. A few sweat tubules are also encircled by sympathetic adrenergic fibers, already described^30^, co-expressing NPY. These fibers are probably responsible for the adrenergic sweating^31–33^ hypothesized to be correlated to psychogenic sweating^16^ or to the metabolic control of glandular growth and plasticity^34^.

### Arrector pili muscles

A detailed description of the autonomic innervation is lacking in humans and animals although a prominent sympathetic adrenergic innervation was previously described^29,35,36^. Our data confirmed that the majority of autonomic fibers in arrector pili muscles are noradrenergic and are likely to be involved in piloerection, but interestingly we found no expression of NPY. This finding suggests that the contingent of sympathetic noradrenergic fibers going to skin contains at least two different subtypes of fibers: one to skin arterioles and sweat glands co-expressing NPY and a different subtype not expressing NPY innervating arrector pili muscles. Sympathetic cholinergic fibers running close to noradrenergic fibers were also found in arrector pili muscles. They are likely involved in piloerection but their specific function is not known. We also disclosed a low number of parasympathetic cholinergic fibers in arrector pili muscles not previously described but, also in this case, their function is unknown and deserves more studies.

### Sensory innervation

A description of sensory fibers in the skin dermis has already been reported^37^ and a previous immunohistochemical study has found a prevalent expression of SP in rat primary sensory neurons^38^ as an indication of their sensory nature. The function of sensory fibers in autonomic skin annexes is difficult to understand but since these fibers are ubiquitously expressed in all skin autonomic skin structures they may be involved in the reflex control of the activity of sympathetic and parasympathetic fibers.

The specific pattern of skin autonomic innervation we described may be potentially useful to interpret the selective damage of skin autonomic fibers in human diseases. Parkinson’s disease is characterized by the highly prevalent involvement of adrenergic fibers going to skin arterioles^9–11^. Based of the data of our study this finding may suggest the involvement of NPY fibers in Parkinson’s disease. By contrast, Ross syndrome^12,13^ shows an absence of sudomotor fibers and a less important decrease of autonomic fibers going to skin vessels and arrector pili muscles in the affected limb. This mirrors the distribution of the skin sympathetic cholinergic fibers we described. However, since we have not had the change to test specific marker of different subtypes of skin autonomic fibers in skin samples from patients with Parkinson’s disease or Ross syndrome further studies are needed to confirm a selective damage in these disorders.

A limitation of this study is the low number of subjects recruited. However, this limitation is counterbalanced by the high number of skin structures in which the innervation has been analysed: 196 including 91 skin arterioles, 47 arrector pili muscles and 58 sweat glands.

In conclusion, hairy skin contains sympathetic (both adrenergic and cholinergic) and parasympathetic fibers co-expressing specific neuropeptides. They are differentially distributed in skin structures. NPY is co-expressed in adrenergic fibers of skin vessels and sweat glands but absent in adrenergic fibers of arrector pili muscles whereas CGRP, VIP and SP are co-expressed in cholinergic fibers both of sympathetic and parasympathetic types. However, the pattern of autonomic innervation we report in the skin cannot be generalized and extended to other organs since non-adrenergic NPY positive fibers were described in the gut^39^ or the thyroid gland^40^. Nevertheless, our data may help to disclose the selective involvement of subtypes of skin autonomic fibers in different human diseases.
